# Thyroid Cancer Incidence around the Belgian Nuclear Sites, 2000–2014

**DOI:** 10.3390/ijerph14090988

**Published:** 2017-08-31

**Authors:** Claire Demoury, Tom De Smedt, Harlinde De Schutter, Michel Sonck, Nancy Van Damme, Kaatje Bollaerts, Geert Molenberghs, Lodewijk Van Bladel, An Van Nieuwenhuyse

**Affiliations:** 1 Scientific Institute of Public Health (WIV-ISP), 1050 Brussels, Belgium; claire.demoury@wiv-isp.be (C.D.); tom.desmedt@p-95.com (T.D.S.); kaatje.bollaerts@p-95.com (K.B.); 2 Belgian Cancer Registry, 1210 Brussels, Belgium; harlinde.deschutter@kankerregister.org (H.D.S.); nancy.vandamme@kankerregister.org (N.V.D.); 3 Federal Agency for Nuclear Control, 1000 Brussels, Belgium; michel.sonck@fanc.fgov.be (M.S.); lodewijk.vanbladel@fanc.fgov.be (L.V.B.); 4 Department of Electronics and Informatics, Vrije Universiteit Brussel, 1050 Brussels, Belgium; 5 Interuniversity Institute for Biostatistics and Statistical Bioinformatics (I-BioStat), Universiteit Hasselt and KU Leuven, 3500 Hasselt and 3000 Leuven, Belgium; geert.molenberghs@uhasselt.be; 6 Department of Public Health and Primary Care, Section Environment and Health, KU Leuven, 3000 Leuven, Belgium

**Keywords:** thyroid cancer, incidence, nuclear sites, ecological study

## Abstract

The present study investigates whether there is an excess incidence of thyroid cancer among people living in the vicinity of the nuclear sites in Belgium. Adjusted Rate Ratios were obtained from Poisson regressions for proximity areas of varying sizes. In addition, focused hypothesis tests and generalized additive models were performed to test the hypothesis of a gradient in thyroid cancer incidence with increasing levels of surrogate exposures. Residential proximity to the nuclear site, prevailing dominant winds frequency from the site, and simulated radioactive discharges were used as surrogate exposures. No excess incidence of thyroid cancer was observed around the nuclear power plants of Doel or Tihange. In contrast, increases in thyroid cancer incidence were found around the nuclear sites of Mol-Dessel and Fleurus; risk ratios were borderline not significant. For Mol-Dessel, there was evidence for a gradient in thyroid cancer incidence with increased proximity, prevailing winds, and simulated radioactive discharges. For Fleurus, a gradient was observed with increasing prevailing winds and, to a lesser extent, with increasing simulated radioactive discharges. This study strengthens earlier findings and suggests increased incidences in thyroid cancer around two of the four Belgian nuclear sites. Further analyses will be performed at a more detailed geographical level.

## 1. Introduction

In August 2008, an incidental gaseous release of I-131 occurred at the Institute for Radio-Elements (IRE) situated in the nuclear site of Fleurus. Following the incident, the Belgian Minister of Social Affairs and Public Health commissioned a study to assess, by means of an epidemiological study at the national level, the possible health risks for populations living in proximity of nuclear power plants or other facilities that can be at the origin of a leak of radioactive material. An ecological study was performed over the period 2000–2008 to assess the possible risk of thyroid cancer for people residing near nuclear sites [1,2]. For two of the four Belgian sites (Mol-Dessel and Fleurus), the incidences of thyroid cancer within the 20-km proximity area were higher than expected.

Exposure to ionizing radiation, particularly during childhood, is the best-established risk factor associated with thyroid cancer [3]. Significantly increased risks for developing thyroid carcinomas following radiation exposure during childhood were observed among people treated by radiotherapy or exposed in nuclear accidents or among survivors of the atomic bombings. For example, a direct consequence of the Chernobyl disaster was an increase in the number of thyroid cancers in children during the years following the accident [4]. Ionizing radiation can also cause thyroid cancer in adults, but will occur after exposures to higher doses [5]. The risk in Japanese A-bomb survivors is marginally elevated [6]. The radionuclide I-131 is known to be a major contributor to thyroid cancer risk in case of nuclear accidents [7].

The present study follows the recommendations resulting from the previous one, which were: “To repeat the epidemiological monitoring within five years, since by that time more cancer data will have become available.” The previous analyses were then replicated for the period 2000–2014. The investigation particularly focused on the question of whether thyroid cancer incidence is higher than expected in the vicinity of the nuclear sites in Belgium. In addition, the hypothesis of a gradient in thyroid cancer incidence with increasing levels of surrogate exposures (proximity, prevailing wind from the nuclear site, and I-131 doses estimated by mathematical modeling) were explored.

## 2. Materials and Methods

### 2.1. Cancer and Population Data

Thyroid cancer incidence data were selected from the Belgian Cancer Registry (BCR), a national population-based registry. Data was available from 2000 to 2014 for the Flemish Region (the northern part of Belgium) and from 2004 to 2014 for the Brussels-Capital Region and the Walloon Region (the southern part of Belgium) at the level of the communes. Belgium has a total of 589 communes, divided over the Flemish Region (*n* = 308; 6,410,705 inhabitants in 2014), Walloon Region (*n* = 262; 3,576,325 inhabitants in 2014), and Brussels-Capital Region (*n* = 19; 1,163,486 inhabitants in 2014). The incidence year, sex, age, and place of residence at diagnosis were used to characterize the cancer diagnosis.

Population counts of the communes by age and sex were obtained from the population registers of the Federal Public Service Economy, Directorate-General Statistics and Economic Information for the period 2000–2014.

### 2.2. Nuclear Sites and Surrogate Exposure

#### 2.2.1. Nuclear Sites

The nuclear sites under study are Doel (Flemish Region), Tihange (Walloon Region), Mol-Dessel (Flemish Region), and Fleurus (Walloon Region), the four Belgian nuclear sites containing facilities of class 1 defined as facilities with the highest radiologic risk [8]. Doel and Tihange are electricity-generating nuclear power plants. The nuclear site of Mol-Dessel primarily consists of the Belgian Nuclear Research Centre (SCK-CEN) and hosts a combination of nuclear activities (applied research and metrology, scientific and technological research, operational waste management). The nuclear site of Fleurus primarily consists of the IRE, one of the major production sites of radioiodines for usage in diagnostic and therapeutic nuclear medicine in Europe. IRE is therefore a major potential source of I-131 emissions (see [2], for a more detailed description of the sites).

#### 2.2.2. Proximity

The proximity area of a nuclear site was constructed as the aggregation of communes having their centroid lying within a circle of 20 km radius centered on the site. As the choice of the proximity area is to a certain extent arbitrary [9] and for comparison with the literature, proximity areas with a 0–5, 0–10, and 0–15 km and with a 5–10, 10–15, and 15–20 km radius were additionally considered.

#### 2.2.3. Prevailing Winds

Data on wind direction and speed between 2003 and 2014 were collected using survey stations by the Belgian Federal Agency for Nuclear Control (FANC). Wind velocity measurements below 0.2 m/s are associated with unstable and continuously changing wind directions and thus were not considered. Wind directions were converted into 16-sector compass roses and based on its centroid, each commune was assigned to a sector. The frequency (in percentage) of the wind blowing from the site toward the sector of the commune was then calculated.

#### 2.2.4. Radioactive Discharges Estimates

I-131 doses to the thyroid (in Sieverts) were calculated for the sites of Mol-Dessel and Fleurus since they showed a significant increasing incidence with distance or prevailing winds, or both (see Results). Estimates were calculated by the FANC with Hotspot (University of California, Lawrence Livermore National Laboratory—UC LLNL, Livermore, CA, USA, Hotspot version 2.07 [10]) assuming standard releases (total activity: 1015 Bq), average meteorological conditions (wind speed: 3 m/s; annual percentage rain fall: 5%), and release height of 80 m for Mol-Dessel and 35 m for Fleurus. The model assumes that dispersion in the upwind and cross-wind direction takes the form of a Gaussian curve, with the maximum concentration in the centre of the plume. The model further assumes that a steady state exists in the radioactive discharges and the meteorological conditions. Finally, the exposure at every commune’s centroid consists of the simulated exposures expressed as a function of distance from the source multiplied by the wind direction frequencies (in %).

### 2.3. Statistical Analyses

#### 2.3.1. Descriptive Analyses

Thyroid cancer incidence was first explored in function of age (5-year age groups), sex, incidence year, and geographical location. The occurrence of thyroid cancer as a function of age and sex was investigated by calculating age- and sex-specific incidence rates. Regional incidences were explored by calculating the age- and sex-standardized rates (European Standard Population). Ninety-five percent confidence intervals (CIs) were calculated by Poisson approximation. In addition, to investigate the thyroid cancer incidence after the Fleurus incident, we calculated the age- and sex-standardized rates for the period 2004–2007 and for the period 2008–2014 in the 0–20 km proximity area around Fleurus, for all ages and for ages less than 20 years. All incidences are expressed as the number of new cases per 100,000 persons per year.

#### 2.3.2. Incidence of Tyroid Cancer around Nuclear Sites

Adjusted Rate Ratios (RRs) were obtained from Poisson regressions. The model included proximity to the nuclear site, age (5-year groups), a quadratic term for age group, sex, incidence year, and region (Walloon and Brussels—Capital Regions vs. Flemish Region). To reflect age and sex differences as well as incidence year and regional differences, interaction terms between age and sex and between incidence year and region were included as well. To account for overdispersion, the model was fitted using the quasi-likelihood approach with the Pearson-based overdispersion parameter [11].

#### 2.3.3. Association between Surrogate Exposures and Incidence of Thyroid Cancer around Nuclear Sites

Surrogate exposures considered were the inverse residential distance from nuclear site, the prevailing winds, and the simulated discharges. To test the hypothesis of a positive gradient in thyroid cancer with increasing levels of each surrogate exposures, three focused hypothesis tests were performed: (a) the conditional form of Stone’s test fixing the total number of cases observed within the proximity area [12]; (b) the conditional form of Bithell’s Linear Risk Score test (LRS) [12] with these exposure as scores θ_i_; and (c) the conditional form of Bithell’s Linear Risk Score test (LRS rank) with corresponding ranks. The advantage of Stone’s test is that it is invariant to monotonic transformations of the “exposure” variable and as such, avoids the need to specify the “exposure”-response relationship. The LRS tests are, compared to Stone’s test, more powerful in case the scores properly reflect the “exposure” effect, but assumptions are made regarding the scale of the “exposure” effect when defining the scores θ_i_. Monte Carlo simulations from the multinomial distribution with 5000 iterations were performed to obtain the *p*-values of the tests.

Finally, the shapes of the exposure–response relationships were investigated. To this end, generalized additive models [13] were used. In particular, the Poisson regression model described above was extended by allowing the previously assumed constant RR to vary smoothly as a function of exposure. The smooth function was taken to be a B-splines basis of 10 B-splines of third degree with a second-order discrete smoothness penalty to control for overfitting [14] (see [1] for a more detailed description of the statistical methodology).

The analyses were generated using SAS software (version 9.3, SAS Institute Inc., Cary, NC, USA) and R software version 3.3.2 (R Core Team, Vienna, Austria) [15].

## 3. Results

### 3.1. Descriptive Analyses

**Figure 1:**
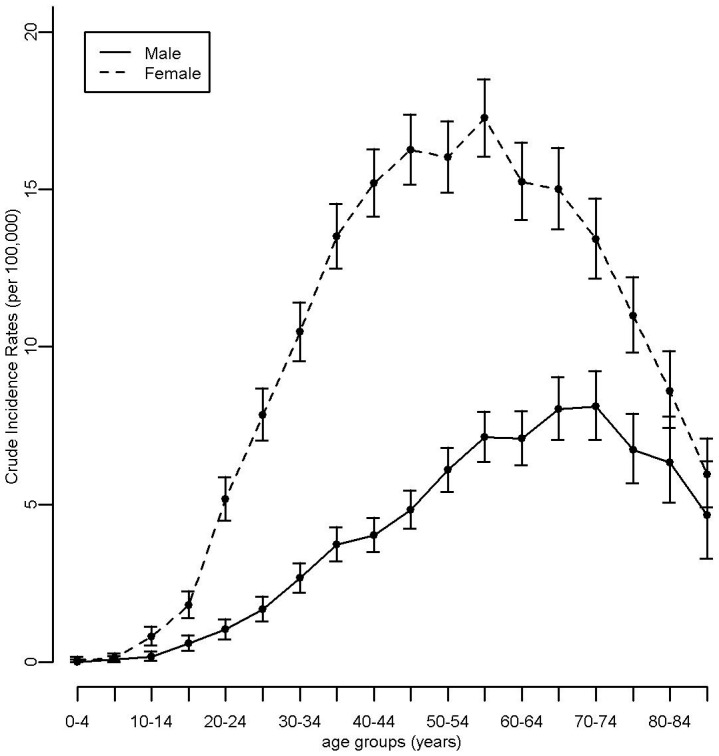
Age- and sex-specific incidence rates of thyroid cancer in Belgium, 2000/2004–2014.

**Figure 2:**
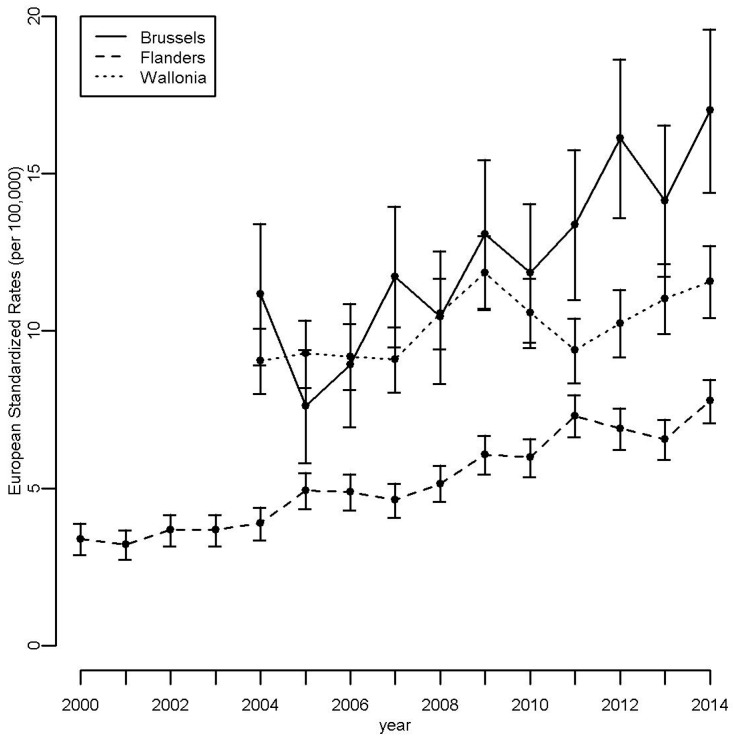
Age- and sex-standardized rates (standardized according to the European Standard Population) of thyroid cancer by year of diagnosis and region in Belgium, 2000/2004–2014.

Overall, 9881 cases of thyroid cancer were registered by the BCR between 2000 and 2014. The age- and sex-specific incidence rates of thyroid cancer are presented in Figure 1. Rates were higher for women than for men, the differences being significant for the ages ranging from 10 to 79 years. Age- and sex-specific incidence rates were higher among middle-aged people. The European standardized incidence rates of thyroid cancer (Figure 2) were significantly lower in the Flemish Region as compared to the Walloon and Brussels-Capital Regions for the whole period. The three Regions showed increasing incidences over time, this trend being significant only in the Flemish Region. In the vicinity of Fleurus, the age- and sex-standardized rates for the periods 2004–2007 and 2008–2014 were not significantly different: resp. 11.1 (95% CI: 9.7–12.5) and 11.5 (95% CI: 10.5–12.6) for all ages, resp. 0.75 (95% CI: 0.01–1.49) and 0.85 (95% CI: 0.26–1.43) for ages less than 20 years.

### 3.2. Incidence of Tyroid Cancer around Nuclear Sites

Table 1 presents RRs of thyroid cancer for the 0–5, 0–10, 0–15, and 0–20 km proximity areas around each nuclear site and the four nuclear sites together. In the vicinity of Doel and Tihange, lower incidences of thyroid cancer were observed, with significant RRs for the 0–10 and 0–20 km proximity areas around Doel and the 0–15 and 0–20 km proximity areas around Tihange. For Mol-Dessel, higher incidences of thyroid cancer were found. Rate ratios ranged from 1.30 (95% CI: 0.97–1.74) for the 0–5 km proximity area to 1.04 (95% CI: 0.94–1.16) for the 0–20 km proximity area, all being borderline not-significant. In the vicinity of Fleurus, results showed slightly increased incidence estimates. RRs were borderline not-significant for the 0–5, 0–10, 0–15, and 0–20 km proximity areas. The analyses around the four Belgian sites together showed slightly increased incidence estimates for the 0–5, 0–10, and 0–15 km proximity areas and a significantly lower rate for the 0–20 km area.

The analyses based on the 5–10, 10–15, and 15–20 km proximity areas around each nuclear site and the four nuclear sites together gave similar results (Supplementary Materials Table S1).

### 3.3. Association between Surrogate Exposures and Incidence of Thyroid Cancer around Nuclear Sites

**Figure 3:**
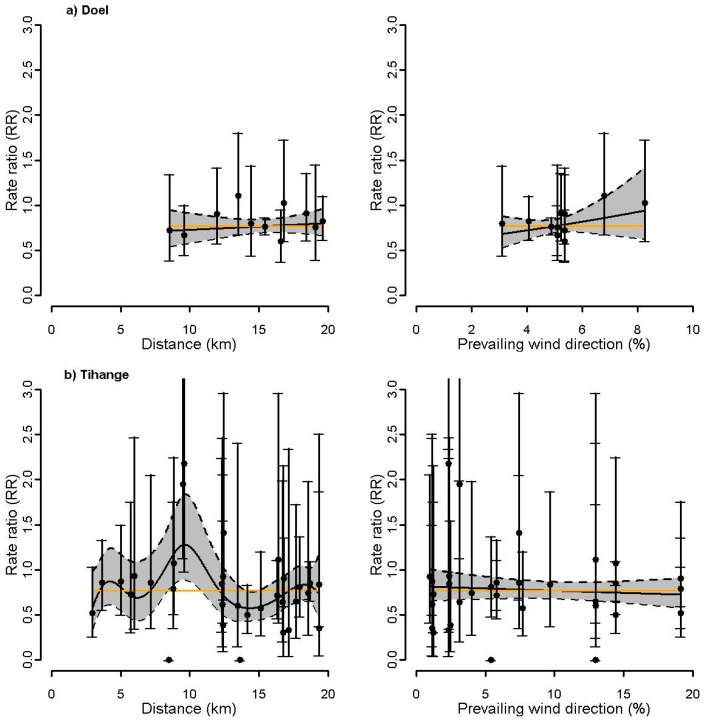
(**a**) Doel; (**b**) Tihange: Rate ratios (RR) and 95% confidence interval (CI) (gray area) of thyroid cancer incidence within the 20 km proximity areas as a smooth function of 1: residential proximity to the nuclear site and 2: prevailing wind directions. The orange line represents the constant RR. The dots represent the commune-specific RRs with their 95% CI.

**Figure 4:**
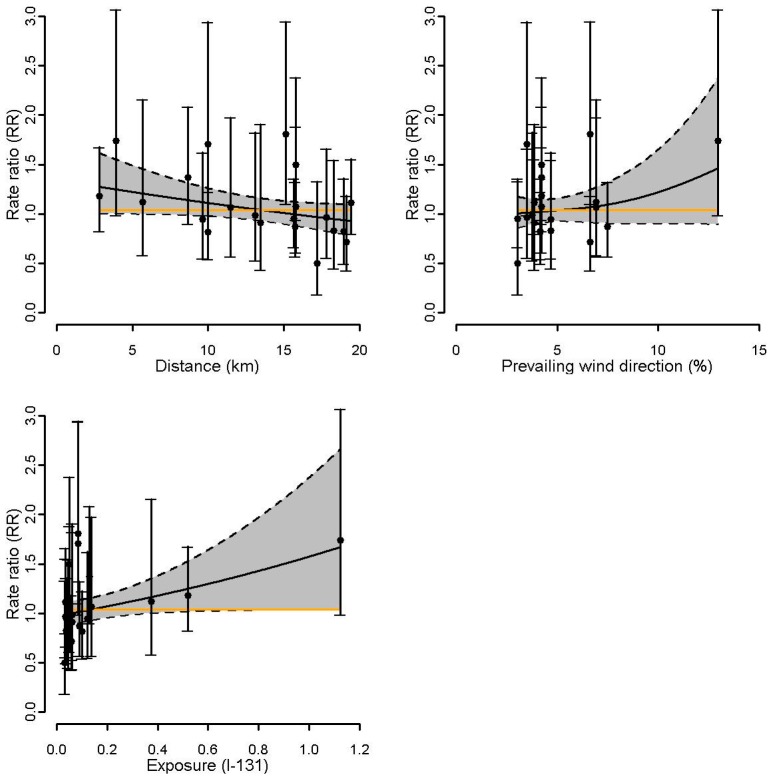
Mol-Dessel: Rate ratios and 95% CI (gray area) of thyroid cancer incidence within the 20 km proximity areas as a smooth function of 1: residential proximity to the nuclear site, 2: prevailing wind directions, and 3: I-131 estimates. The orange line represents the constant RR. The dots represent the commune-specific RRs with their 95% CI.

**Figure 5:**
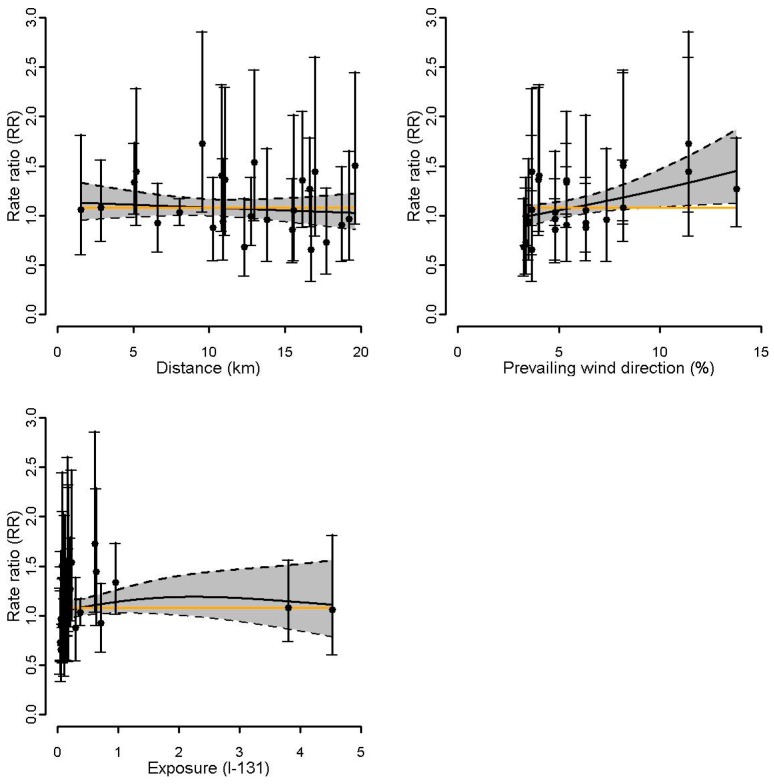
Fleurus: Rate ratios (RR) and 95% CI (gray area) of thyroid cancer incidence within the 20 km proximity areas as a smooth function of 1: residential proximity to the nuclear site, 2: prevailing wind directions, and 3: I-131 estimates. The orange line represents the constant RR. The dots represent the commune-specific RRs with their 95% CI.

For the site of Doel (Figure 3a), the estimated exposure response curve using residential proximity did not suggest a gradient for thyroid cancer incidence, whereas a positive gradient was observed for wind direction. The p-values of the focused tests (Stone, LRS, and LRS rank test), however, were not significant (Table 2). For Tihange (Figure 3b), both the estimated exposure response curves and the focused tests did not show any evidence for gradients (Table 2). For Mol-Dessel, Figure 4 is indicative of an increasing gradient in thyroid cancer with increasing proximity, increasing prevailing dominant wind from the site, and increasing exposure to I-131. For proximity, the focused tests showed significant *p*-values (LRS: 0.04, LRS rank: 0.05) or a small *p*-value (Stone test: 0.10) (Table 2). For wind direction, the *p*-values of the focused tests were small and ranged from 0.11 to 0.19. For exposure to I-131, the *p*-values of the Stone test, LRS, and LRS rank were 0.06, 0.03, and 0.08, respectively. For the site of Fleurus, Figure 5 did not suggest a gradient of the estimated exposure response curve using residential proximity, but is indicative of an increasing gradient in thyroid cancer with increasing prevailing wind direction. This was confirmed by the focused hypothesis tests: the *p*-values for proximity were not significant, whereas the *p*-values of the focused tests for wind direction were significant (0.01 for the three tests) (Table 2). For exposure to I-131, Figure 5 did not clearly suggest a gradient of thyroid cancer incidence. The focused tests however showed small to significant *p*-values (Stone test: 0.10, LRS: 0.08, LRS rank: 0.03) (Table 2).

## 4. Discussion

The present study investigated the incidence of thyroid cancer around the four Belgian nuclear sites at the level of the communes over the period 2000–2014. No increased incidence of thyroid cancer was observed around the nuclear power plants of Doel and Tihange. The focused tests and the estimated exposure response curves did not suggest a gradient for thyroid cancer incidence. In contrast, increases in thyroid cancer incidence were found around the nuclear sites of Mol-Dessel and Fleurus, but risk ratios were not significant. For Mol-Dessel, the focused tests and the estimated exposure response curves showed a gradient for thyroid cancer incidence, irrespective of the surrogate exposure (proximity, prevailing wind direction frequency, or radioactive discharge estimates). For Fleurus, a gradient for thyroid cancer incidence was observed with increasing prevailing dominant wind from the site and less clearly with increasing exposure to I-131.

This study is a follow-up study of Bollaerts et al. 2014 [2] and Bollaerts et al. 2015 [1] over a prolonged period. In Bollaerts et al. 2014, over the period 2000–2008, no increased thyroid cancer incidence was found within the 20 km proximity area around the nuclear power plants of Doel and Tihange. For the sites of Mol-Dessel and Fleurus, the incidences of thyroid cancer within the 20 km proximity area were higher than expected [2]. The present 15-years follow up study confirms the previous results. For Mol-Dessel, the results of the focused hypothesis tests and estimated exposure-response curves were far from significant [1]. The present study which includes a larger number of cases over a longer period showed evidence for a gradient in thyroid cancer incidence with surrogate exposures (proximity, prevailing winds, and radioactive discharges). In the past analyses, for Fleurus, the focused hypothesis tests suggested an increased incidence using radioactive discharge estimates as surrogate and less clearly using prevailing winds [1]. This is in line with the results of the present study.

We used three measures as surrogate exposures leading to different assumptions and limitations. The first measure was the residential proximity to the source, assuming that the risk of thyroid cancer is decreasing monotonously from the source and that this decrease is independent of the direction (isotropic). The second measure was the prevailing wind direction frequency, assuming that the risk of thyroid cancer is higher when the wind is blowing more often towards the residential commune. The distance is hence not taken into account. Finally, we have further combined both the effect of distance and the effect of wind by modeling the estimated discharges from the plants. This method is the radio-ecologically most plausible, but the potential bias of measurement error may be more pronounced because it combines distance and wind direction misclassifications. In addition to the different surrogates of exposure, different statistical methods were exploited. In particular, three different focused hypothesis tests (i.e., Stone’s test, Bithell’s LRS test with levels of surrogate exposure, and Bithell’s LRS test with ranks) were used, all ranging differently with respect to the trade-off between power and the need to correctly specify the exposure–response relationship. The use of different statistical methods reduces the dependence of the results on the tests assumptions. In the present study, conclusions were strengthened since the methods and globally, the surrogate exposures give similar results. The nuclear power plants of Chooz and Borssele are situated close to the Belgian border, and parts of Belgian territory are within the 20 km proximity area around these installations. Both sites were not included in the present study. The Belgian territory around the nuclear site of Borssele was not included because no Belgian commune has its centroid within the 20 km proximity area. Some Belgian communes have their centroid within the proximity area of Chooz. However, previous analyses showed that results were unstable because of the small population size [2].

In the present study, data are compared at the population-level rather than at the individual level so that conclusions cannot be transferred at the individual level. We used cancer incidence data aggregated at the level of the commune (ecological design). Data in the Belgian Cancer Registry are available for the year of cancer diagnosis and the place of residence where the incident case lives at the moment of diagnosis. Hence, measurement error could have occurred because migration phenomena were not taken into account. In general, the misclassification of exposure due to migration is assumed to be non-differential. The aggregation of data causes a loss of information that could lead to an ecological bias. Ecological bias arises from the inability of ecological data to characterize within-area variability in confounders and exposures [16]: we did not account for individual characteristics which could potentially confound the associations. Moreover, due to the size of the geographical areas, exposure misclassification may arise. Further analyses will be performed at the level of the statistical sectors, the smallest basic areal unit defined by the Federal Public Service Economy, Directorate-General Statistics and Economic Information for which population data and cancer cases become available. Such analyses would reduce ecological bias due to within-area variation of the exposure.

The increased thyroid cancer incidences were observed around Mol-Dessel and Fleurus, the two nuclear sites with research and industrial activities, and not around the nuclear power plants. These differences could be associated with differences in exposure or risk between the two types of sites. Fleurus is one of Europe’s major production sites of radio-iodines. Regarding the level of exposure during the incident (International Nuclear and Radiological Event Scale, INES-rating: 3), an estimated gaseous amount of 48 GBq of I-131 was released to the environment. Regarding earlier exposures, the post-incident investigation indicated points of serious concern with regard to both the operational safety and the management of the Fleurus’ site [2]. Radio-iodines releases before the incident cannot be ruled out. The Mol-Dessel site, in combining research and industrial nuclear and radiological activities, is and always has been characterized by a great variety of possible radioisotope releases, radio-iodines being one of them. Significant releases of radio-iodines have never been reported to the authorities, but cannot be completely excluded, in particular before the 1990s, when an active surveillance network was not yet available. Illegal radioactive waste storage and treatment activities have taken place in the past in Dessel and might have been at the origin of unauthorized historical releases.

Most of the studies on thyroid cancer incidence and nuclear sites investigated the risk for thyroid cancer among residents living near nuclear power plants. Knowledge about the degree of risk nevertheless remains uncertain. A recent review and meta-analysis found no increased risk of thyroid cancer associated with living near nuclear power plants [17]. The pooled estimates did not reveal different patterns of risk by gender, exposure definition, or reference population. However, sensitivity analysis by exposure definition showed that living less than 20 km from nuclear power plants was associated with a significant increase in the risk of thyroid cancer in well-designed studies (summary OR = 1.75; 95% CI = 1.17–2.64). Thyroid cancer risk caused by radioactive discharges from nuclear power plants might be too small to be statistically detectable. Although the thyroid is one of the organs most sensitive to ionizing radiation, the measured radiation dose has been reported to be very low near nuclear power plants under normal operating conditions in most countries [18].

It was of interest to investigate whether the observed incidence pattern could be compatible with levels of radiation exposure from the sites. However, these levels are often below the detection limit of the routine environmental monitoring in Belgium, by which natural background radiation is predominantly measured. Therefore, we considered emission data and used a model for computing I-131 radioactive discharges estimates since measurements were not available. It should be noted that it is based on strictly hypothetical releases.

A substantial increase in papillary thyroid carcinoma among children exposed to the radioiodine fallout has been one of the main consequences of the Chernobyl reactor accident [4]. Recently, the investigation of papillary thyroid carcinoma from a cohort revealed an overexpression of the *CLIP2* gene among young patients exposed to the post-Chernobyl radioiodine fallout at a very young age [19]. More recently, mechanistic models were proposed to explore the impact of radiation on the molecular landscape of papillary thyroid carcinoma [20,21]. The findings point to a function of *CLIP2* as a driver gene in radiation-induced papillary thyroid carcinoma. These models constitute a potential promising interface between molecular biology and radiation epidemiology.

The incidental gaseous release of I-131 at the IRE in Fleurus occurred in 2008. Whereas the study period of the previous study [1,2] stopped at the time of the incident, the time window of the available data now allows for the investigation of the health effects of the Fleurus 2008 incident itself. The age- and sex-standardized rates in the 0–20 km proximity area of Fleurus over the periods 2004–2007 and 2008–2014 were not significantly different. Nevertheless, it should be noted that radiation-induced thyroid cancers are characterized by a long latency period, and individual variation in latency time exists [22]. Thyroid cancer in young children is characterized by a shortened latency period, between 10 and 15 years [23,24], and in the very young age (neonates, babies, toddlers), latency could be some five years or even less. However, the thyroid cancer incidence among people less than 20 years of age is not sufficiently high to capture a potential beginning of increase, and the age- and sex-standardized rates in the 0–20 km proximity area of Fleurus over the periods 2004–2007 and 2008–2014 were not significantly different.

## 5. Conclusions

This study strengthens earlier findings and suggests increased incidences in thyroid cancer around the nuclear sites of Mol-Dessel and Fleurus, the two nuclear sites with research and industrial activities. Further analyses will be performed at a more detailed geographical level.

## Figures and Tables

**Table 1:** Rate ratios of thyroid between 2000 (2004) and 2014 for the 0–5, 0–10, 0–15, and 0–20 km proximity area around each nuclear site and the four Belgian nuclear sites together.

RR ^a^					
Nuclear Site	PY	O	E	Est.	95% CI
Doel ^b^ (2000–2014)					
0–5 km	-	-	-	-	-
0–10 km	953,438	34	49.02	0.68	(0.49, 0.95)
0–15 km	1,891,550	80	98.10	0.81	(0.65, 1.00)
0–20 km	11,582,195	462	590.88	0.77	(0.70, 0.84)
Tihange ^c^ (2004–2014)					
0–5 km	375,054	28	38.57	0.72	(0.50, 1.03)
0–10 km	951,538	87	98.26	0.88	(0.72, 1.08)
0–15 km	1,546,391	125	160.05	0.77	(0.65, 0.92)
0–20 km	3,368,078	274	350.06	0.77	(0.68, 0.87)
Mol-Dessel ^b^ (2000–2014)					
0–5 km	632,633	43	32.95	1.30	(0.97, 1.74)
0–10 km	2,045,060	123	105.79	1.15	(0.97, 1.37)
0–15 km	2,569,901	150	132.68	1.12	(0.96, 1.31)
0–20 km	6,285,927	340	323.53	1.04	(0.94, 1.16)
Fleurus ^c^ (2004–2014)					
0–5 km	368,755	41	38.26	1.07	(0.80, 1.44)
0–10 km	3,493,371	392	359.07	1.10	(0.99, 1.21)
0–15 km	4,767,612	529	492.32	1.08	(0.99, 1.18)
0–20 km	6,204,514	685	640.77	1.08	(1.00, 1.17)
All sites					
0–5 km	1,376,442	112	109.77	1.02	(0.86, 1.22)
0–10 km	7,443,407	636	612.14	1.05	(0.97, 1.13)
0–15 km	10,775,454	884	883.15	1.01	(0.95, 1.08)
0–20 km	27,440,714	1761	1905.23	0.91	(0.87, 0.95)

PY: person-years at risk; O: observed number of cases; E: expected number of cases; Est.: estimate; 95% CI: 95% Wald confidence interval. ^a^ RR: rate ratios adjusted for age, sex, incidence year and region; ^b^ Flemish Region as reference region; ^c^ Walloon/Brussels-Capital Region as reference region.

**Table 2:** Results (*p*-values) of the Bithell’s Linear Risk Score test (LRS), Bithell’s Linear Risk Score test with corresponding ranks (LRS rank) and Stone test for measures of surrogate exposure in the vicinity of the nuclear sites (≤20 km).

	Proximity ^a^			Wind ^b^			I-131 ^c^		
	Stone	LRS	LRS Rank	Stone	LRS	LRS Rank	Stone	LRS	LRS Rank
Doel	0.81	0.68	0.65	0.31	0.19	0.26	-	-	-
Tihange	0.45	0.45	0.52	0.80	0.71	0.69	-	-	-
Mol-Dessel	0.10	0.04	0.05	0.11	0.19	0.18	0.06	0.03	0.08
Fleurus	0.35	0.25	0.24	0.01	0.01	0.01	0.10	0.08	0.03
All	<0.01	<0.01	<0.001	0.51	0.45	0.38	-	-	-

^a^ Residential proximity to nuclear site; ^b^ Prevailing winds; ^c^ Radioactive discharge estimates based on mathematical modeling.

## Supplementary Materials

The following are available online at www.mdpi.com/1660-4601/14/9/988/s1, Table S1: Rate ratios of thyroid between 2000 (2004) and 2014 for the 0–5, 5–10, 10–15, and 15–20 km proximity area around each nuclear site and the four Belgian nuclear sites together.
