# Supplementary material for: Thyroid Cancer Incidence around the Belgian Nuclear Sites, 2000–2014
Source: Int J Environ Res Public Health. 2017 Aug 31;14(9):988. doi: 10.3390/ijerph14090988 (PMC5615525; doi:10.3390/ijerph14090988)
Supplement: Supplementary file 1 [file ijerph-14-00988-s001.pdf]

Table S1. Rate ratios of thyroid between 2000 (2004) and 2014 for the 0–5, 5–10, 10–15, and 15–20 km proximity area around each nuclear site and the four Belgian nuclear sites together.

**Distance to the Nuclear Site (km)**	**PY**	**O**	**E**	**RR [a]**	
				**Est.**	**95% CI**
Doel [b] (2000-2014)					
(0–5)	-	-	-	-	-
(5–10)	953,438	34	49.02	0.68	(0.49,0.95)
(10–15)	938,112	46	49.08	0.93	(0.70,1.24)
(15–20)	9,690,645	382	492.77	0.77	(0.69,0.85)
Tihange [c] (2004-2014)					
(0–5)	375,054	28	38.57	0.72	(0.50,1.03)
(5–10)	576,484	59	59.69	0.99	(0.77,1.27)
(10–15)	594,853	38	61.79	0.61	(0.45,0.83)
(15–20)	1,821,687	149	190.01	0.78	(0.66,0.91)
Mol-Dessel [b] (2000-2014)					
(0–5)	632,633	43	32.95	1.30	(0.97,1.74)
(5–10)	1,412,427	80	72.84	1.09	(0.88,1.35)
(10–15)	524,841	27	26.89	0.99	(0.69,1.43)
(15–20)	3,716,026	190	190.85	0.98	(0.85,1.13)
Fleurus [c] (2004-2014)					
(0–5)	368,755	41	38.26	1.07	(0.80,1.44)
(5–10)	3,124,616	351	320.81	1.10	(0.99,1.22)
(10–15)	1,274,241	137	133.25	1.03	(0.87,1.21)
(15–20)	1,436,902	156	148.45	1.05	(0.90,1.23)
All sites					
(0–5)	1,376,442	112	109.77	1.02	(0.86,1.22)
(5–10)	6,066,965	524	502.36	1.05	(0.97,1.14)
(10–15)	3,332,047	248	271.01	0.93	(0.82,1.04)
(15–20)	16,665,260	877	1022.09	0.84	(0.79,0.89)

PY: person-years at risk; O: observed number of cases; E: expected number of cases; Est. : estimate; 95% CI: 95% Wald confidence interval. [a] RR: rate ratios adjusted for age, sex, incidence year and region; [b] Flemish Region as reference region; [c] Walloon/Brussels-Capital Region as reference region.
